# Chemoprofiling of *Himantoglossum robertianum* (Loisel.) P. Delforge leaves reveals predominance of gastrodigenin and structurally related compounds

**DOI:** 10.1007/s13659-025-00526-7

**Published:** 2025-07-16

**Authors:** Ilaria Chiocchio, Antonio De Agostini, Manuela Mandrone, Pierluigi Cortis, Clarissa Tarozzi, Ferruccio Poli, Cinzia Sanna

**Affiliations:** 1 Department of Pharmacy and Biotechnology (FaBit), Alma Mater Studiorum, University of Bologna, Via Irnerio 42, 40126 Bologna, Italy; 2 Department of Life and Environmental Sciences, University of Cagliari, Via S. Ignazio da Laconi 13, 09123 Cagliari, Italy

**Keywords:** *Himantoglossum robertianum*, NMR metabolomics, Orchids, *Gastrodia elata*, Parishin, 4-hydroxybenzyl alcohol

## Abstract

**Purpose:**

The purpose of this study was to phytochemically profile *Himantoglossum robertianum* leaves. In fact, despite its wide distribution and its use in traditional medicine, this orchid is still understudied and little is known about its phytochemicals.

**Methods:**

The analyses were performed by ^1^H NMR fingerprinting, elucidated by further 2D NMR and UHPLC-MS experiments. Both primary and secondary metabolites were qualified and quantified. The study was carried out comparing six natural populations by metabolomics approach, allowing further considerations on the influence of the environment on the concentration of metabolites.

**Results:**

This work brings to light a surprising phytochemical parallel between *H. robertianum* and the medicinal orchid *Gastrodia elata.* In fact, the most abundant specialized metabolites resulted: gastrodigenin, gastrodin, bis(4-hydroxybenzyl)ether, parishin A, parishin C, and parishin E. Interestingly, these metabolites are all known for their potential in the treatment of neurological disorders and are, indeed, the active principles of *Gastrodia elata*, an important orchid used in Traditional Chinese Medicine. The active metabolites were present in all the natural populations, where only slight variations in their concentration were revealed.

**Conclusion:**

Mapping the metabolome of *H. robertianum* leaves has provided new insights into the study of orchids, including diagnostic signals for rapid identification of gastrodigenin-like compounds directly from the ^1^H NMR profile of a crude extract. From a bioprospecting perspective, finding active metabolites in leaves makes the plant source more valuable than the perennial hypogeal organs that are usually the herbal drug of orchids (i.e. *G. elata*).

**Graphical Abstract:**

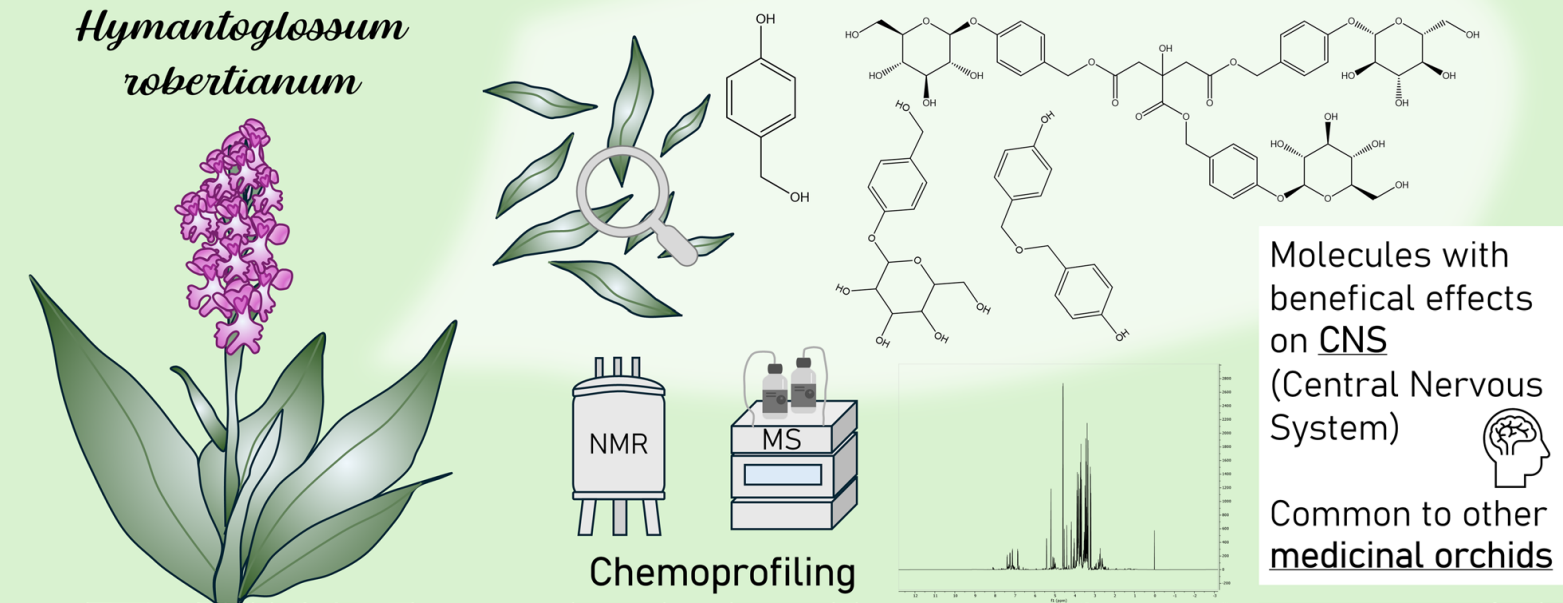

**Supplementary Information:**

The online version contains supplementary material available at 10.1007/s13659-025-00526-7.

## Introduction

The considerable number of species, together with the ecological plasticity and diversity, make the Orchidaceae family a promising taxon for bioprospecting and searching for compounds of biological interest [1, 2]. Nevertheless, the knowledge of orchids' metabolome significantly expands our understanding of evolution, biodiversity, and conservation [1].

**Fig. 1:**
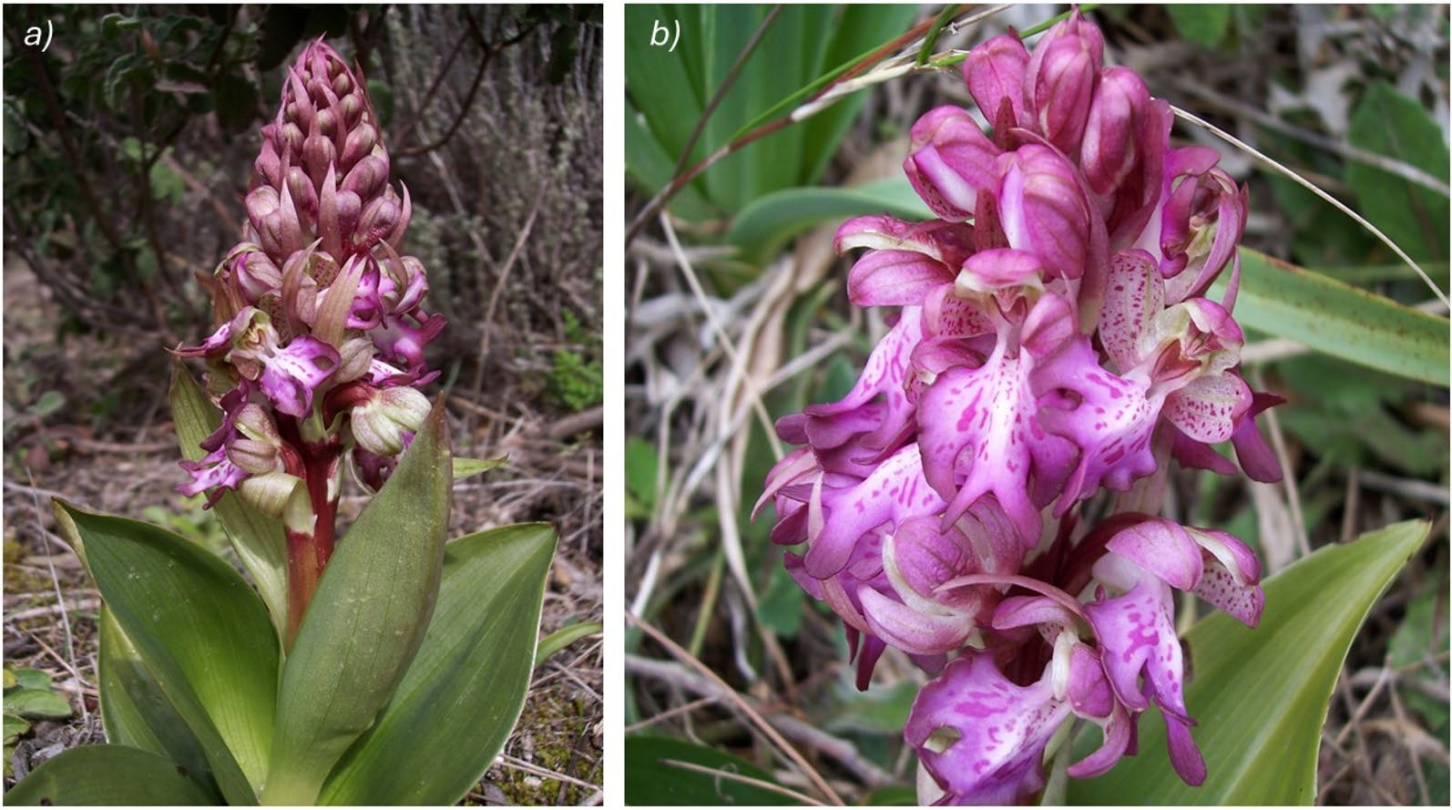
*Himantoglossum robertianum* in its natural habitat (**a**) and a detail of its inflorescence (**b**) (photos by C. Sanna)

*Himantoglossum* Spreng (1826) is a genus of the Orchidaceae family, widely spread across the Mediterranean basin, from Portugal to Anatolia [3]. Its species are known for their striking appearance and substantial biomass production, as well as for the use in the production of the traditional beverage “Salep” across Asia Minor, Germany, Greece, Iran, Afghanistan, and India [4]. While on one hand, *Himantoglossum* species have been extensively studied through ecological and molecular phylogenetic research [5, 6], on the other hand, the genus is still underexplored in terms of phytochemical profile. *H. robertianum* (Loisel.) P. Delforge (syn. *Barlia robertiana*), among the other species of the same genus, is scarcely studied. It is a Mediterranean orchid thriving in grasslands, garrigues, and open woodlands, generally on alkaline substrates under dry to moist conditions up to 1700 m above sea level (a.s.l.) [7]. Due to its adaptability *H. robertianum* can be easily found in roadside verges and degraded urban lots [5]. Frequently referred to as the “giant orchid” because of its dimensions ranging from 25 to 80 cm, this plant features thick stems and a basal rosette composed of 5–10 leaves. It stands as one of the earliest blooming orchids in Italy, and its inflorescences are dense and reach heights between 6 and 23 cm, carrying up to 60 flowers [7] (Fig. 1).

Noteworthy, *H. robertianum* hypogeum organs are used in different Mediterranean regions as food supplements, tonic, aphrodisiac, and to treat coughs and gastric diseases [8, 9].

However, as premised, the phytochemical knowledge about this species is scant at the date. The primary focus of the scientific research has been on its flowers, analyzed for their volatile profiles [10–12], phenolic composition, and bioactivities [13]. Only Badalamenti et al. [14] analyzed *H. robertianum* bulbs and roots, finding two bioactive dihydrophenanthrenes (loroglossol and hircinol), in an apolar chloroform extract of these hypogeum organs, leaving still unknown the widest overview of their phytochemical composition. Moreover, no phytochemical studies have been conducted on the leaves of this species.

Metabolomics and, more generally, metabolite fingerprinting (chemoprofiling) of plant extracts are gaining increasing importance in plant science. In this context, the combined use of different analytical platforms is strongly encouraged, as they can provide a comprehensive overview of the metabolome, revealing phytochemical diversity and assisting in the discovery of unique or bioactive metabolites, as well as chemotaxonomic markers. Among the available analytical techniques, ^1^H NMR spectroscopy stands out for its robustness, reproducibility, and minimal sample preparation requirements. It provides a wide range of both primary and specialized metabolites, along with their relative concentrations [15]. Additionally, ^1^H NMR allows the acquisition of raw data that can be easily shared, reused, or integrated into metabolomic databases, facilitating the development of chemometric models and predictive tools.

Within this framework, the objective of this study was to elucidate the unknown phytochemical profile of *H. robertianum* leaves by means of NMR spectroscopy supported by MS experiments, providing, for the first time, a comprehensive ^1^H NMR metabolite fingerprint of *H. robertianum* leaves, and taking also into account the possible variations among six different populations in Sardinia (Italy).

## Results

### Leaf metabolite fingerprinting

**Fig. 2:**
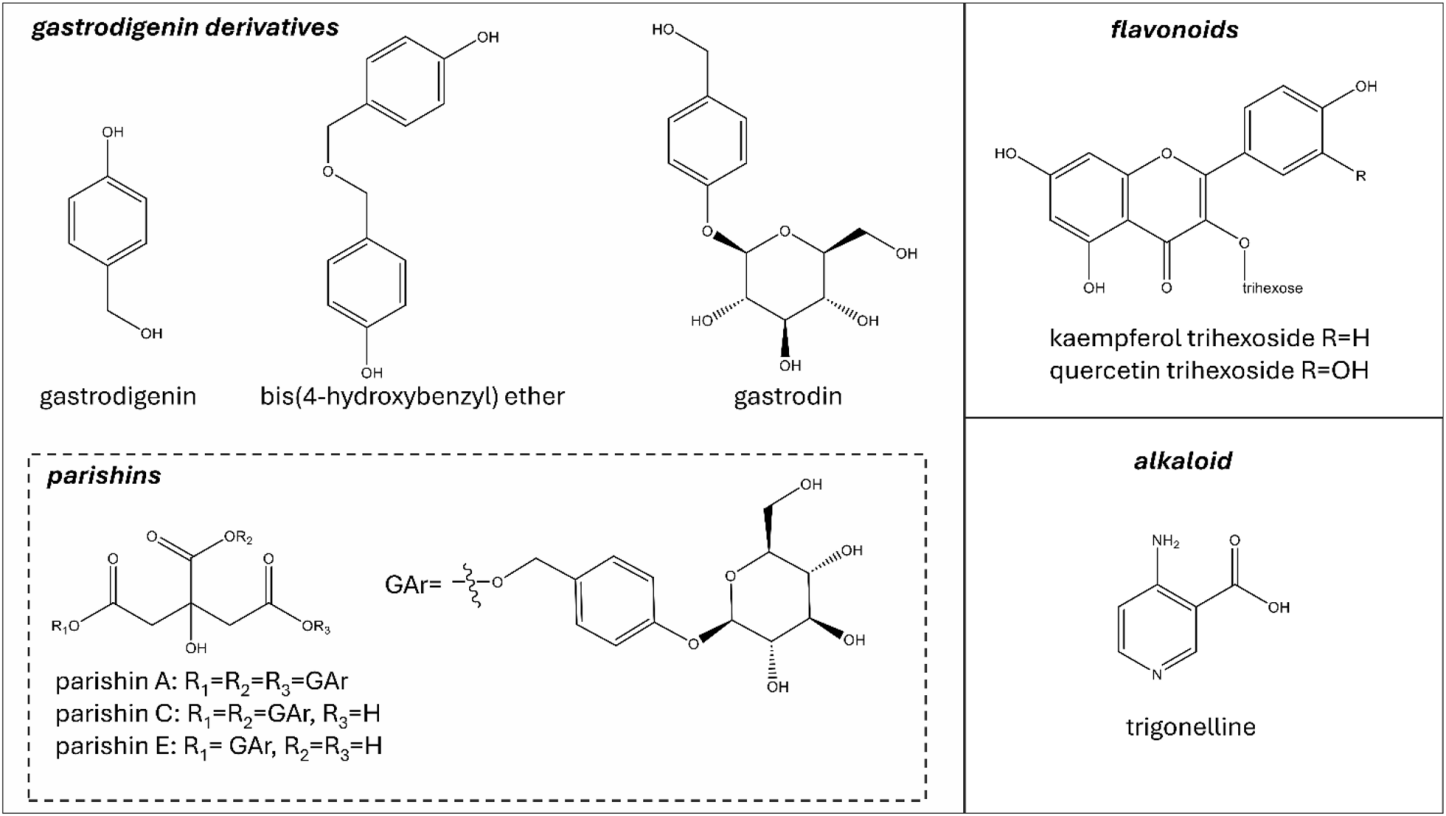
Secondary metabolites found in *H. robertianum* leaves: the gastrodigenin-related compounds are shown in the square on the left. Flavonoids stereochemistry has not been explicated since they were annotated only by UHPLC-MS

The fractionation procedure (see experimental section) and the analyses carried out by NMR, UHPLC-UV/ESI–MS, and ESI-QToF-MS on *H. robertianum* leaves extract led to the identification of several gastrodigenin-related compounds and two flavonoids (Fig. 2).

**Fig. 3:**
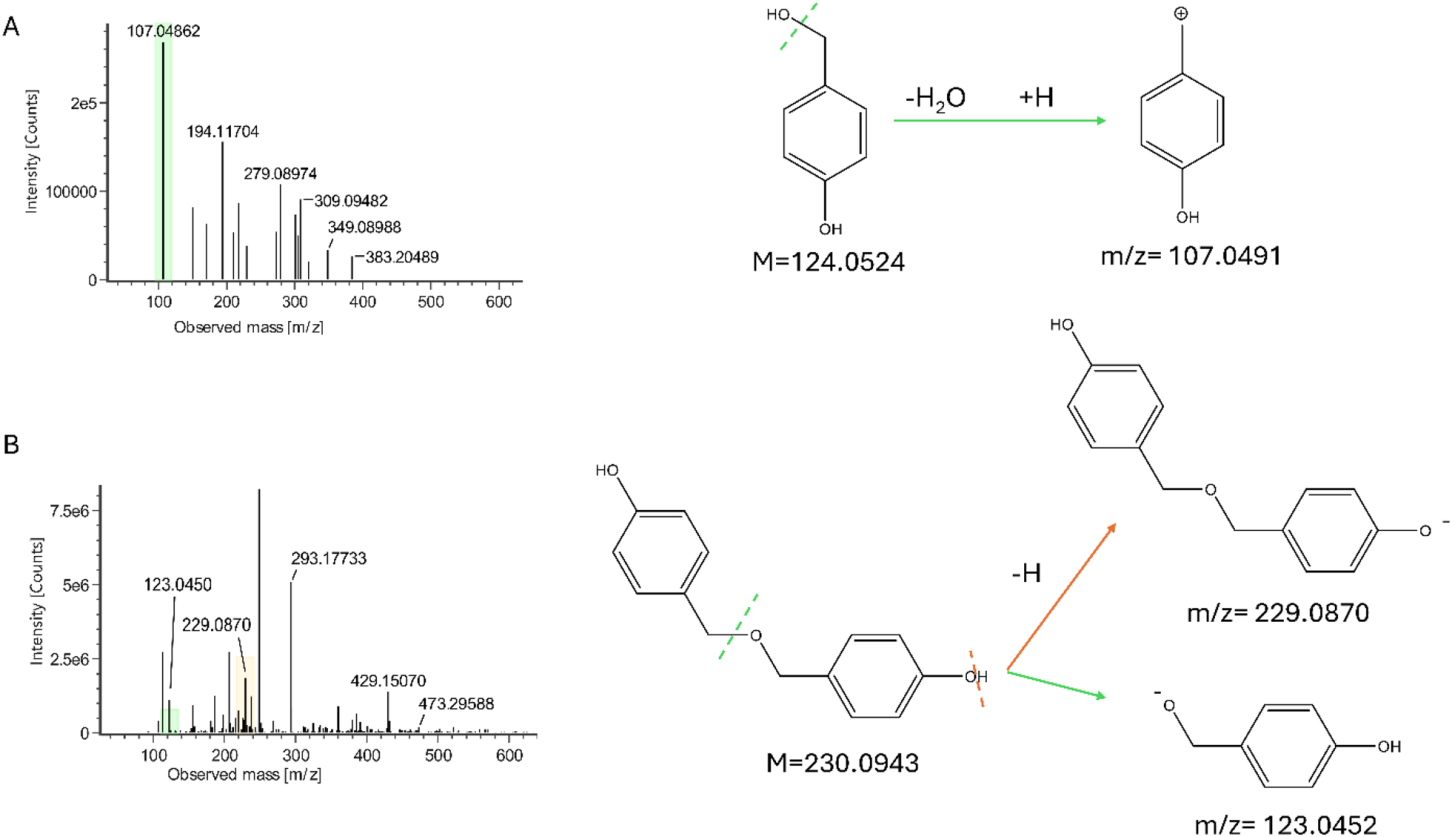
Mass spectra and proposed fragmentation of the compounds, showing theoretical values of (**A**) FrEt subfraction 10 containing gastrodigenin, and (**B**) FrEt subfraction 22 containing bis(4-hydroxybenzyl)ether

Gastrodigenin and bis(4-hydroxybenzyl)ether (Fig. 2) were separated by preparative medium-pressure liquid chromatography (MPLC) performed on the ethyl acetate fraction (FrEt) obtained by liquid/liquid partition of the raw extract. Despite the different polarity of the two compounds (indicated by their elution time), they both gave the *m/z* value 107 when analyzed by UHPLC-UV/ESI–MS in positive mode. Moreover, the NMR spectra (mono and bidimensional) of the two compounds appeared very similar, showing the typical features of the 4-hydroxy-benzilic moiety. The ESI-QToF-MS analysis (Fig. 3) was conclusive in the identification of gastrodigenin in one fraction and the bis(4-hydroxybenzyl) ether in the other fraction.

Some challenges also concerned the identification of parishin A (known as parishin), which was pre-purified by MPLC performed on the water fraction. In this case, the repetition of three identical moieties within the molecules (Fig. 2) caused several overlapping signals in the NMR spectra, making the information provided by the MS analysis necessary to elucidate the molecular structures. Easier was the structure elucidation of parishin E, found in another fraction pre-purified by MPLC. The full structural elucidation and key correlations in NMR of parishin A and E are shown in Figure S1 and Figure S2, respectively (chemical shift of protons and carbons are given in the materials and methods section).

**Fig. 4:**
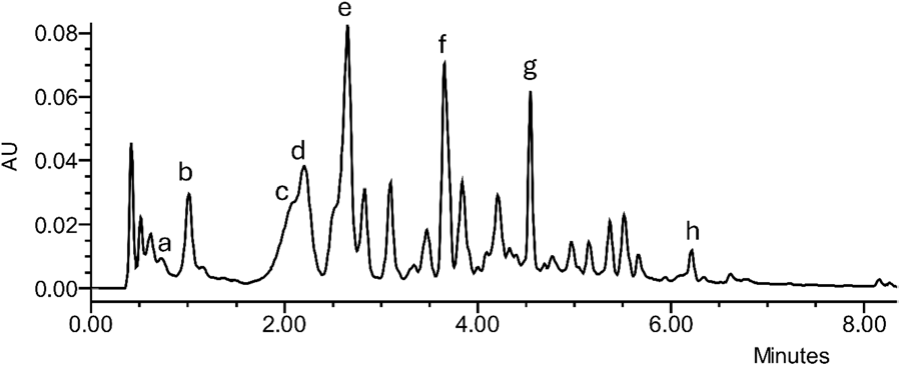
Explorative chromatogram at λ 268 nm of a representative sample (from SUP location), a = gastrodin, b = gastrodigenin, c = parishin E, d = quercetin trihexoside, e = kaempferol trihexoside, f = parishin C, g = parishin A, h = bis (4-hydroxybenzyl) ether. Interpretation of the relative MS spectra is reported in Table 1

**Fig. 5:**
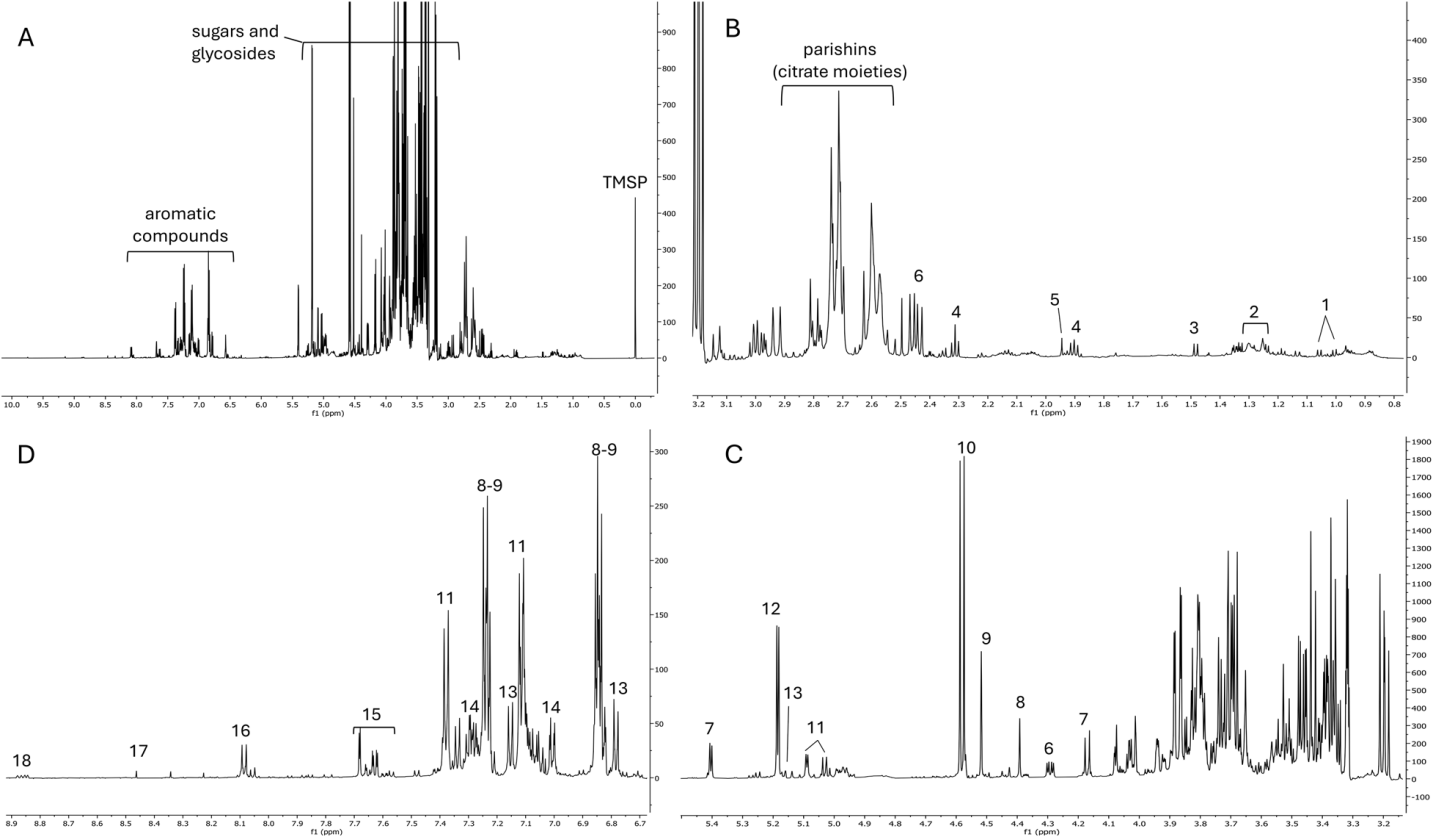
^1^H NMR profile of a representative sample (from SIS location). (**A**) Total spectrum; (**B**) region from δ 0.8 to 3; (**C**) region from *δ* 3.6 to 5.5; **D** region from *δ* 6.7 to 8.9; 1 = valine; 2 = fatty acids; 3 = alanine; 4 = GABA; 5 = acetic acid; 6 = malic acid; 7 = sucrose; 8 = bis(4-hydroxybenzyl)ether; 9 = gastrodigenin; 10 = β-glucose; 11 = parishin E; 12 = α-glucose; 13 = putative parishin C; 14 = parishin A; 15 = quercetin trihexoside; 16 = kaempferol trihexoside; 17 = formic acid; 18 = trigonelline

The overall fractionation and structural elucidation procedure paved the way to understand that several compounds bearing a gastrodigenin moiety were present in *H. robertianum* leaves, and therefore, they shared a common ion in our system, namely the fragment 107 in positive mode (interpreted as C_7_H_7_O^+^). On this basis, we profiled the raw extract by UHPLC-UV/ESI–MS analysis (Fig. 4), following the elution at *λ* 268 nm (typical of *p*-substituted aromatic rings and parishins) and targeting the *m/z* value 107. As expected, we found this *m/z* value under multiple chromatographic peaks at different retention times (RTs), some of which were generated by the compounds already identified, and others could be attributed to further putative gastrodigenin-related compounds. In particular, at RT 0.8 min, co-eluting with the ion 107, also the *m/z* value 309 was found in positive ionization mode, interpreted as gastrodin [M + Na]^+^ (Fig. 2, Table 1). Moreover, at RT 3.6 min, co-eluting with the ion 107 (positive mode), the *m/z* value 727 was found in negative mode, consistent with a parishin bearing two gastrodin moieties, which was tentatively identified as parishin C [M-H]^−^ (Fig. 2, Table 1), corroborated also by the ^1^H NMR profiling (see compound 13 of Fig. 5).

**Table 1:** Experimental *m/z* values (both in positive and negative ionization mode) of the secondary metabolites putatively identified by Mass Spectrometry

Compound name	RT (min)	Molecular formula	Experimental *m/z*		Interpreted adduct
Gastrodin	0.8	C_13_H_18_O_7_	ESI^+^	107 309	C_7_H_7_O^+^ [M + Na]^+^
			ESI^−^	331	[M + HCOO]^−^
Gastrodigenin*	1	C_7_H_8_O_2_	ESI^+^	**107.0491** 107	[M-H_2_O + H]^+^ [M-H_2_O + H]^+^
Parishin E*	2.1	C_19_H_24_O_13_	ESI^+^	107 478	C_7_H_7_O^+^ [M + NH_4_]^+^
			ESI^−^	459	[M-H]^−^
Quercetin trihexoside	2.2	C_33_H_40_O_22_	ESI^+^	789	[M + H]^+^
Kaempferol trihexoside	2.6	C_33_H_40_O_21_	ESI^+^	773	[M + H]^+^
			ESI^−^	817	[M + HCOO]^−^
Parishin C	3.6	C_32_H_40_O_19_	ESI^+^	107 751	C_7_H_7_O^+^ [M + Na]^+^
			ESI^−^	727	[M-H]^−^
Parishin A*	4.5	C_45_H_56_O_25_	ESI^+^	107 1014	C_7_H_7_O^+^ [M + NH_4_]^+^
			ESI^−^	1041 **995.30483** **1041.3093**	[M + HCOO]^−^ [M-H]^−^ [M + HCOO]^−^
bis (4-hydroxybenzyl) ether*	6.1	C_14_H_14_O_3_	ESI^+^	107	C_7_H_7_O^+^
			ESI^−^	275 **123.0450** **229.0870**	[M + HCOO]^−^ [M/2-H]^−^ [M-H]^−^

Bold values were obtained by ESI-QToF-MS. *Partially purified and fully characterized also by NMR spectroscopy

The same UHPLC analysis performed at *λ* 370 nm, a wavelength typical for flavonoid detection, showed two main peaks attributed to quercetin trihexoside and kaempferol trihexoside (Fig. 2, Table 1).

The overall work on structural elucidation and annotation helped to characterize the ^1^H NMR fingerprint of *H. robertianum* leaves (Fig. 5), which itself permitted the identification of additional metabolites, such as the alkaloid trigonelline and several primary metabolites, including sugars (*α*-glucose, *β*-glucose, and sucrose), protein amino acids (valine, alanine, threonine) the nonprotein amino acid GABA, and organic acids (malic acid, acetic acid, formic acid).

**Fig. 6:**
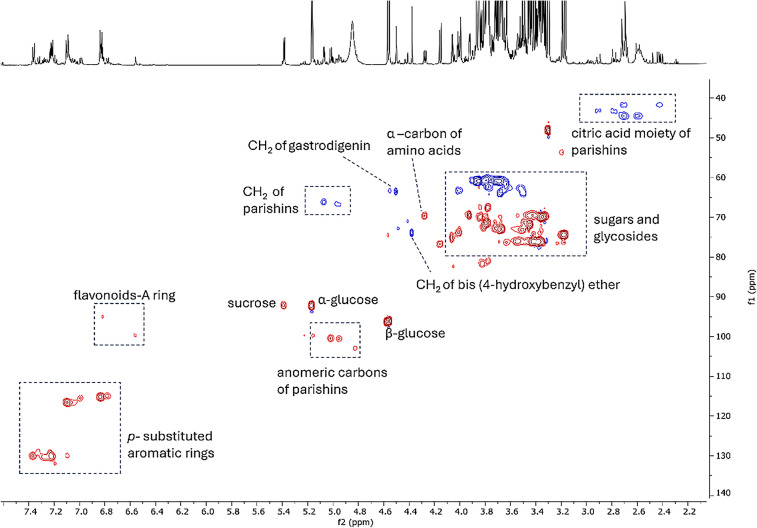
HSQC-DEPT spectrum of the raw extract (SIS location) highlighting diagnostic signals of the most abundant compounds (red color shows the ^1^H-^13^C correlations in CH_3_ and CH groups, while the blue color shows the ^1^H-^13^C correlations in CH_2_ groups)

The most abundant metabolites with their ^1^H NMR diagnostic signals were further confirmed by HSQC experiment performed on the raw leaf extract (Fig. 6).

### Metabolomic analysis of the populations

The compounds identified in *H. robertianum* leaf were semi-quantified by comparison with the internal standard in ^1^H NMR profiling (Table 2). Moreover, considering that plants vary their metabolome in response to environmental factors, we refined our investigation on *H. robertianum* by metabolomic analysis of 6 populations grown in different locations (Table 2).

**Table 2:** ^1^H NMR based semi-quantitative analysis of the metabolites detected in *H. robertianum* from six locations of Sardinia

	NMR signal used for the semi-quantification	Sant’Isidoro (SIS) μg/mg of leaf DW	Rio Bau Onu (BAO) μg/mg of leaf DW	Capo Mannu (CAM) μg/mg of leaf DW	Domusnovas (DOM) μg/mg of leaf DW	Jerzu (JER) μg/mg of leaf DW	Su Planu (SUP) μg/mg of leaf DW
*Primary metabolites*							
Alanine	*δ* 1.47, d	0.34 ± 0.04	0.14 ± 0.04	0.19 ± 0.02	0.23 ± 0.04	0.21 ± 0	0.02 ± 0
Acetic acid	*δ* 1.95, s	0.11 ± 0.03	0.02 ± 0.01	0.07 ± 0	0.06 ± 0.01	0.04 ± 0	0.04 ± 0
α-Glucose	*δ* 5.2, d	84.82 ± 4.05	91.89 ± 14.74	92.14 ± 1.81	108.93 ± 12.34	109.26 ± 3.58	107.53 ± 9.72
β-Glucose	*δ* 4.6, d	145.65 ± 5.94	165.12 ± 30.09	154.22 ± 5.04	191.01 ± 5.16	181.03 ± 3.61	189.04 ± 1.12
Formic acid	*δ* 8.45, s	0.04 ± 0.01	0.03 ± 0	0.03 ± 0.01	0.02 ± 0	0.01 ± 0.01	0.02 ± 0
Gaba	*δ* 1.9, t	1.25 ± 0.12	0.52 ± 0.1	0.64 ± 0.04	0.5 ± 0.15	0.93 ± 0.01	0.31 ± 0.01
Malic acid	*δ* 4.3, dd	16.77 ± 0.69	23.15 ± 11.41	16.3 ± 0.72	21.37 ± 0.34	8.99 ± 0.21	15.27 ± 0.19
Sucrose	*δ* 5.4, d	42.18 ± 1.62	87.42 ± 23.26	78.71 ± 1.6	93.78 ± 2.36	89.25 ± 2.14	78.36 ± 19.11
Valine	*δ* 1.05, d	0.29 ± 0.03	0.16 ± 0.06	0.2 ± 0.01	0.18 ± 0.06	0.16 ± 0	0.23 ± 0
*Specialized metabolites*							
Bis (4-hydroxybenzyl) ether	*δ* 4.39 s	8.21 ± 1.62	11.95 ± 1.68	9.1 ± 0.41	9.81 ± 0.48	9.92 ± 0.29	10.68 ± 0.21
Gastrodigenin	*δ* 4.39, s	13.53 ± 0.56	8.25 ± 1.01	11.91 ± 0.42	12.82 ± 0.36	10.79 ± 0.18	10.11 ± 0.03
Kaempferol trihexoside	*δ* 8.08, d	9.14 ± 0.36	12.58 ± 4.54	9.61 ± 0.3	7.88 ± 0.44	9.74 ± 0.25	7.07 ± 0.24
Parishin A	*δ* 7.29, d	24.11 ± 2.75	65.44 ± 3.22	24.45 ± 0.96	23.05 ± 0.84	27.92 ± 2.48	41.55 ± 1.18
Parishin C	*δ* 6.78, d	9.55 ± 1.01	10.32 ± 0.51	9.53 ± 0.68	6.45 ± 0.23	6.59 ± 0.34	9.63 ± 0.19
Parishin E	*δ* 5.09, d	24.17 ± 0.21	21.79 ± 4.39	19.72 ± 0.78	12.21 ± 0.66	31.89 ± 0.29	22.44 ± 0.34
Quercetin trihexoside	*δ* 7.68, d	14.67 ± 1.22	10.55 ± 7.47	6.84 ± 0.23	1.68 ± 0.4	4.7 ± 0.16	6.96 ± 0.23
Trigonelline	*δ* 8.9, m	0.14 ± 0.09	0.31 ± 0.32	0.14 ± 0.01	0.34 ± 0.01	0.17 ± 0.01	0.13 ± 0.02

Each value is a mean of 3 values (obtained from the 3 individuals harvested in each location) with their standard deviation, and are expressed as μg/mg of DW (dry wight) of leaf. Chemical shift and multiplicity of the diagnostic signals used for the semi-quantification are also given

*d* doublet, *dd* double doublet, *m* multiplet, *s* singlet, *t* triplet

**Fig. 7:**
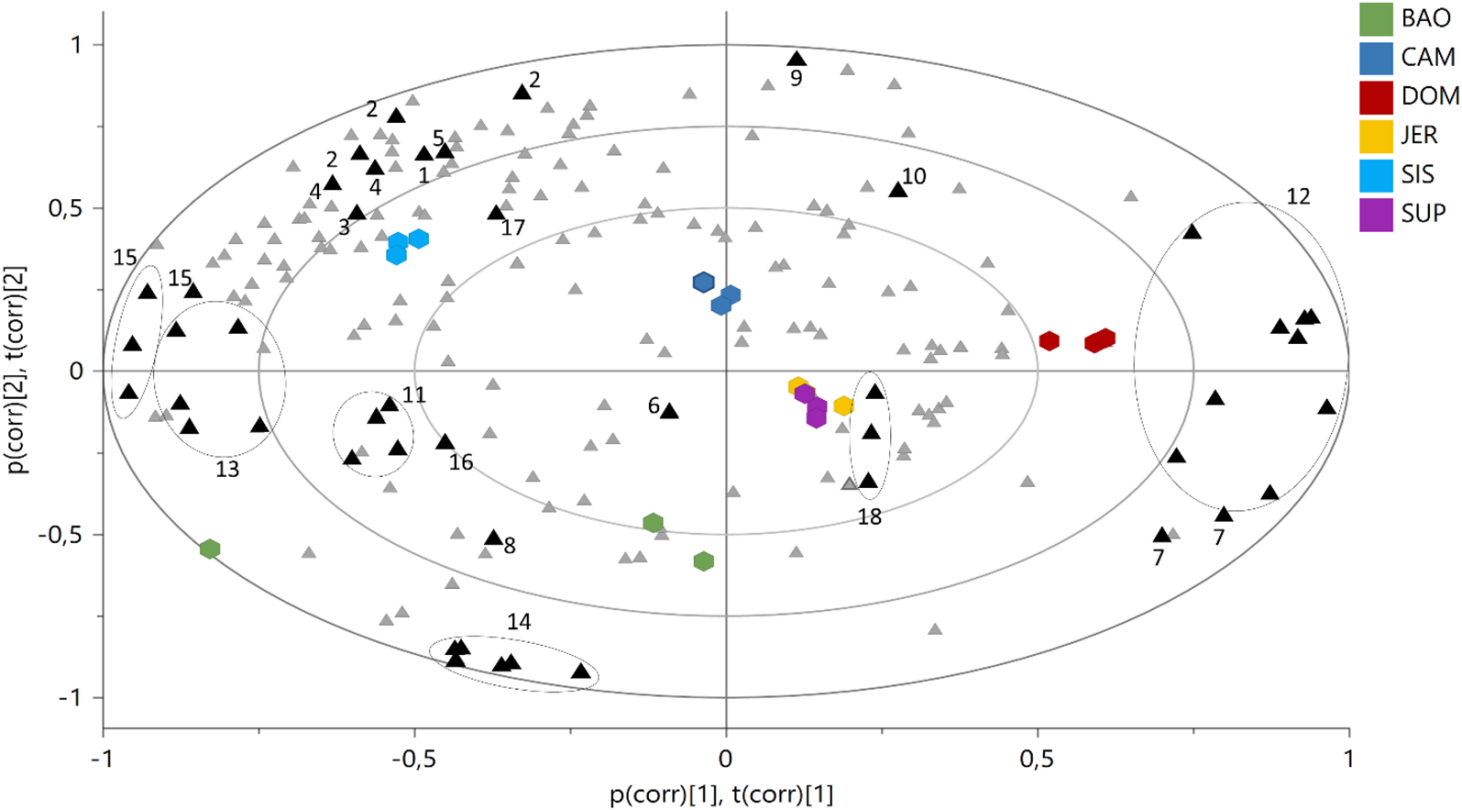
Bi-Plot of the Principal Component Analysis (PCA) based on ^1^H NMR spectra. Coloured hexagons represent individuals from different populations, while triangles are ^1^H NMR buckets, in black are highlighted the buckets related to the diagnostic spectral signals of the identified metabolites. 1 = valine; 2 = fatty acids; 3 = alanine; 4 = GABA; 5 = acetic acid; 6 = malic acid; 7 = sucrose; 8 = bis(4-hydroxybenzyl)ether; 9 = gastrodigenin; 10 = *β*-glucose; 11 = parishin E; 12 = *α*-glucose; 13 = parishin C; 14 = parishin A; 15 = quercetin trihexoside; 16 = kaempferol trihexoside; 17 = formic acid; 18 = trigonelline

For metabolomics, bucketed ^1^H NMR spectra were subjected to Principal Components Analysis (PCA) (Fig. 7) and interpreted according to the ecological distribution of the samples.

Eight Principal Components (PCs) explained a maximum of 98% of the variation in the data set (given by R^2^*x*(cum)), while the obtained Q^2^(cum) was 84%, indicating very good predictability (Q^2^ must be equal or higher than 50%). PC1 and PC2 explained the 41.8% and 24.8% of the variance, respectively.

The shift of the samples along the positive PC1 (i.e. DOM population) was representative of an increasing concentration of *α*-glucose, *β*-glucose, and sucrose, and, on the other hand, of a depletion of all the other metabolites. On the opposite side of the plot, along the negative PC1, were placed the samples characterized by increased concentration of flavonoids (quercetin trihexoside, kaempferol trihexoside), and gastrodigenin-related compounds, such as bis(4-hydroxybenzyl)ether, parishin E, and parishin C. This was the case of the populations of SIS and BAO, which, however, differed for their placement on the PC2. In particular, SIS shifted along the positive PC2, for its highest content of aliphatic amino acids (alanine, valine, threonine), fatty acids, GABA, acetic and formic acid. On the other hand, the position of BAO on the negative PC2 was determined by the highest content of parishin A.

## Discussion

This study is the first phytochemical investigation of *Himantoglossum robertianum* leaf. As a result, we found that the most abundant and characterizing metabolites are gastrodigenin and other structurally related compounds, namely: bis(4-hydroxybenzyl)ether, gastrodin, parishin A, parishin C, and parishin E (Fig. 2). Surprisingly, these findings revealed a phytochemical similarity between *H. robertianum* and *Gastrodia elata* Blume, a medicinal orchid renowned for its extensive use in traditional Chinese medicine (TCM). In fact, the metabolites found in *H. robertianum* leaves are considered the major constituents and bioactive compounds of *G. elata* rhizome [16, 17]. *G. elata* is a saprophytic orchid having no leaves, and whose hypogeal organs are traditionally used for treating neuralgic and nervous diseases, such as epilepsy, paralysis, headaches, and dizziness, as well as for memory enhancement [18]. In support of the traditional uses of this plant, there are several experimental studies available (both in vivo and in vitro), documenting the beneficial effects of its extracts and/or metabolites [18]. In particular, various neuroprotective properties, including protection against cerebral ischemia, have been attributed to gastrodigenin [19–21], and its glycoside gastrodin [22], as well as to parishins [23, 24]. In addition, all of these molecules have been investigated for their cognitive enhancing effects and as promising compounds for the treatment of neurodegenerative and CNS-related diseases [18, 22, 25, 26]. Furthermore, studies have demonstrated a strong binding affinity between Parishin A and the anti-aging protein Klotho, which is known to mitigate aging-related diseases [27].

Conversely, bis(4-hydroxybenzyl)ether appears to be scarcely investigated, with the only reported study by Pyo et al. (2004) [28] describing it as a platelet aggregation inhibitor.

Gastrodigenin and gastrodin are not exclusive to orchids and have been found in several plant species, belonging to a wide range of families [29–34]. Parishins are not that common; they have been found only in *G. elata*, and more recently in *Maclura tricuspidata* (family Moraceae) [35]. Bis(4-hydroxybenzyl)ether has been found only in *G. elata* and two other species of the Orchidaceae family, namely *Pleione yunnanensis* [36] and *Eulophia nuda* [37], both of which also produce gastrodigenin and are used in different traditional medicine systems.

**Fig. 8:**
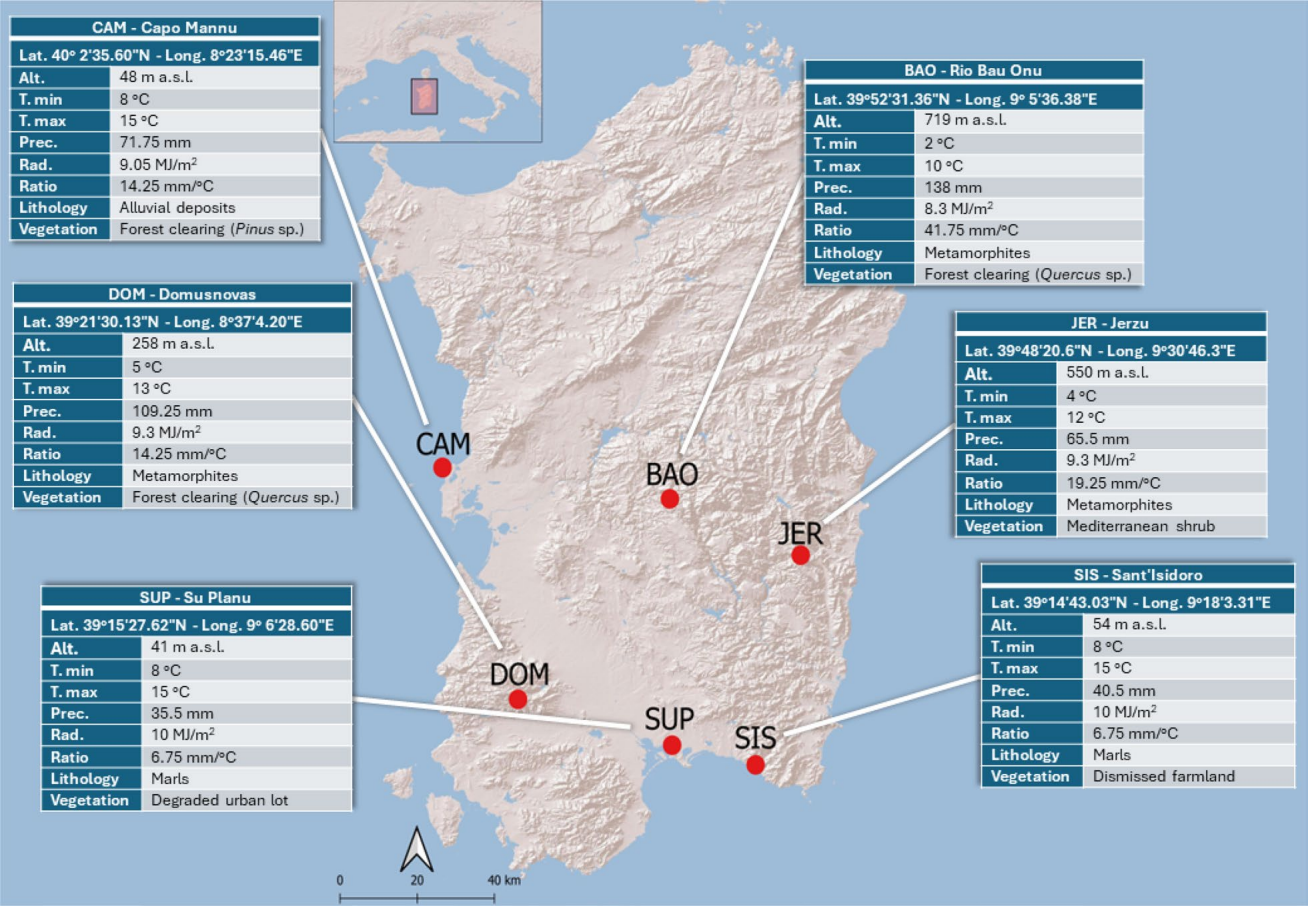
Sampling sites are reported as red dots together with the acronyms of the localities; the informative panels display the localities’ extended names along with the coordinates and the environmental variables considered in the present study: altitude (Alt., m a.s.l.); mean value of the daily temperature minimum (T. min, °C); mean value of the daily temperature maximum (T. max, °C); precipitations (Prec., mm); solar radiation (Rad., MJ/m^2^); precipitation to temperature ratio (Ratio, mm/°C); lithological framework (Lithology); Vegetation

The presence of important medicinal compounds, such as the gastrodigenin-related metabolites in *H. robertianum* draws attention to this understudied orchid. Furthermore, the additional content of flavonoids may contribute to confer antioxidant, immunomodulatory, and anticancer activities [38]. Aware of the influence exerted by the ecosystem on the plant metabolite content, we made our work more complete by studying *H. robertianum* from six different natural populations (Fig. 8), and performing the classic metabolomic workflow based on comparisons, together with statistics on the semi-quantified metabolites.

Importantly, this approach allowed us to definitively conclude that all the 18 analyzed individuals possessed the compounds of pharmaceutical interest. However, the range of concentrations of these metabolites depends on the population, suggesting that *H. robertianum* has developed diverse metabolic strategies to adapt to its local environment.

In particular, BAO, the population growing at the highest altitude (719 m a.s.l.) and exposed to the lowest temperatures, had the highest content of parishin A and the lowest content of gastrodigenin, as highlighted by both PCA (Fig. 7) and ANOVA test (Figure S3).

In addition, the PCA showed that as gastrodigenin increased, the metabolites structurally related to it (parishins and bis(4-hydroxybenzyl)ether) decreased (Fig. 7), supporting the hypothesis that gastrodigenin may be their biosynthetic precursor. This is an important clue, considering that the biosynthetic pathway of parishins is still unexplored.

In terms of metabolome, another extreme case was DOM, a population accumulating a high amount of carbohydrates to the detriment of the majority of the secondary metabolites. In plants, the activation of a few biosynthetic pathways in favor of the production and storage of sugars could be indicative of a quiescent stage in the plant’s life cycle. Despite irradiance value for DOM being uniform with those of the other localities, DOM samples were collected on a north-facing slope, within a *Quercus* sp. forest clearing where the sun reached the understory filtered by tree canopy and this different exposition to sun radiation could have played a role in the quiescent-like state suggested by the metabolome features.

Noteworthy was also the SIS population, which was characterized by a general high content of secondary metabolites and by the highest content of aliphatic amino acids (alanine, valine, threonine), fatty acids, GABA, acetic, and formic acid. These metabolites are typically associated with stress responses and metabolic adjustments to varying environmental conditions [39]. In our study, low precipitations and high mean values of the daily temperature in SIS could partially explain the increasing amount of these metabolites in the population.

A direct correlation between the metabolome of the populations and the monitored environmental variables was not found. It is reasonable to consider that additional factors, especially of biotic origin, which were not monitored in our work, might have an impact on the metabolome.

In this context, it is worth noting that we found in all samples the alkaloid trigonelline. This compound suggests a possible influence of mycorrhizal symbiotic association in the metabolite’s synthesis. It is known that in legumes, trigonelline is accumulated in cotyledons and leaves, translocated to roots and nodules, and degraded to be used as a nutrient source for bacteria growth during all the stages of the symbiotic relation [40]. Actually, Orchidaceae are well known to form unique symbiotic relationships with some soil fungi [41], which offer several benefits to the host plant by stimulating seed germination and growth, improving their resistance to biotic and abiotic stresses, and promoting the production of specialized metabolites [10, 41].

In conclusion, the identification of gastrodigenin and its structurally related compounds from the leaves of *H. robertianum* paves the way for the potential use of this species as a source of valuable therapeutic compounds. Importantly, from the hypothetical perspective of large-scale use of the plant, the presence of the compounds of interest in the leaves suggests an easier and more sustainable exploitation of the species compared to the use of hypogeal (perennial) organs used in other medicinal orchids, such as *G. elata*.

In this context, it is also worth noting that the possibility of the *in-vitro* propagation of the species has been documented [42], making *H. robertianum* a suitable reservoir of promising bioactive compounds avoiding the direct exploitation of the natural resource.

The results of this work invite further studies on the *Himantoglossum* genus, which could present interesting metabolic features, also important to better understand its adaptation and conservation strategies. In this regard, we believe that the elucidated ^1^H NMR profile here provided, based on a few milligrams of plant material, will be helpful for the rapid identification of gastrodigenin-related compounds in other orchids, and could be reused as a metadata for further work, for instance for an orchid metabolome database implementation.

## Material and methods

### Sampling of plant material

The sampling of plant material occurred during the blooming season (February and March) of 2021 as described in De Agostini et al. [10]. In fact, while reports indicate anthesis occurring as early as in November in coastal regions of the southern distribution, the peak bloom typically occurs during the winter months from January to April.

Leaves of *H. robertianum* were gathered from six distinct localities across Sardinia Island (Italy) (Fig. 8), characterized by diverse ecological features. Sampling sites were chosen taking into account the results of our previous study, where the variability of volatile organic compounds (VOCs) produced by *H. robertianum* inflorescences was explored [10].

The sampling sites will be referred in the text as BAO, CAM, DOM, JER, SIS and SUP. The labels refer to the toponymy of the sampling sites: BAO = Bao Onu (municipality of Laconi); CAM = Capo Mannu (municipality of Cabras); DOM = Domusnovas; JER = Jerzu; SIS = Sant’Isidoro (municipality of Quartucciu); SUP = Su Planu (municipality of Selargius). Coordinates, altitude, climatic conditions, lithology, and vegetation types are summarized in Fig. 8 (map obtained by Qgis Software and further modified on Microsoft PowerPoint).

Climatic data are from the climatic monitoring authority of Sardinia (data available at https://www.sar.sardegna.it/pubblicazioni/riepiloghimensili/mensili.asp) and consider the mean values of January and February 2021 as they reflected the climate conditions during plant development. Lithological information derived from Aru et al. [43, 44]. Vegetation data were based on authors’ observations. *H. robertianum* leaves were sampled from three individuals per location. The leaves were carefully stored in zip-locked polyethylene bags containing silica gel for transport, and freeze-dried upon arrival at the laboratory. They were then powdered using an electrical grinder (1 g of fresh material yielded 0.1 g of freeze-dried powder). Specimens (one for each population) were deposited in the *Herbarium* CAG of the Department of Life and Environmental Sciences of the University of Cagliari, with the specimens’ vouchers CAG1305/V1a-f, as already reported in De Agostini et al. [10].

### Chemicals

Deuterium oxide (D_2_O, 99.90% D) and deuterated methanol (CD_3_OD, 99.80% D) were purchased from Eurisotop (Cambridge Isotope Laboratories, Inc, France). Standard 3-(trimethylsilyl)-propionic-2,2,3,3-*d*_*4*_ acid sodium salt (TMSP), sodium phosphate dibasic anhydrous and sodium phosphate monobasic anhydrous and all the other chemicals and solvents were purchased from Sigma-Aldrich Co. (St. Louis, MO, USA).

### Extracts preparation for ^1^H-NMR profiling

Thirty mg of powdered freeze-dried leaves per individual were extracted with 1 mL of mixture (1:1) of phosphate buffer (90 mM; pH 6.0) in D_2_O (containing 0.01% TMSP) and CD_3_OD by ultrasonication (TransSonic TP 690, Elma, Germany) for 20 min. After this procedure, samples were centrifuged for 5 min (17000 × *g*), and then 700 μL of supernatant were transferred into NMR tubes.

### NMR analysis and data treatment

^1^H NMR, homonuclear (COSY and J resolved) and heteronuclear 2D correlation experiments (HMBC, HSQC) were recorded at 25 °C on a Bruker Avance Ascend 600 instrument equipped with autosamplers and a cryoprobe Prodigy. For ^1^H NMR profiling, the instrument operated at ^1^H NMR frequency of 600.13 MHz, and CD_3_OD was used as internal lock. Each ^1^H NMR spectrum consisted of 46 scans with a relaxation delay (RD) of 2 s and spectral width of 9595.8 Hz (corresponding to *δ* 16.0), the measurement lasted 4 min. A presaturation sequence (PRESAT) was used to suppress the residual water signal at *δ* 4.83.

The spectra were manually phased, baseline corrected, and calibrated to the internal standard trimethyl silyl propionic acid sodium salt (TMSP) at *δ* 0.0, this was also used as a standard for semiquantitative analysis. Spectral intensities (in the region from *δ* 0.0 to 10.0), were reduced to the integrated regions of equal width (*δ* 0.04) and normalized by total area using the NMR MestReNova 12 software (Mestrelab Research, Spain).

### Multivariate data analysis and statistics

The obtained data matrix was subjected to multivariate data analysis using the software SIMCA P + (v. 18, Sartorius), the models were built using Pareto scaling. The spectral regions between *δ* 5–4.5 and *δ* 3.34–3.30 were excluded from the analysis because of the residual solvent signals. Metabolites were identified on the basis of literature data [45, 46], in-house database, and further phytochemical analysis. All the spectra are available on Zenodo repository (10.5281/zenodo.14960480).

Values of metabolite concentration (calculated by semi-quantitative analysis) were expressed as μg/mg (leaf DW) as mean and standard deviation (SD) of three individuals. Statistical analyses were performed using Graph Pad Prism 4 software (La Jolla, CA, USA) by one-way analysis of variance (ANOVA), followed by Tukey’s honestly significant difference (HSD) post-hoc test, considering significant differences at P values < 0.05.

### Pre-purification procedures

Pre-purification procedures were carried out to characterize the most abundant secondary metabolites, whose structures could not be elucidated just from the NMR profile. In order to extract preferentially the specialized metabolites, 6.7 g of freeze-dried leaves (obtained by pooling material from all samples) were extracted with 90 mL of MeOH/H_2_O (70:30), filtered on Büchner funnel, and dried in rotary evaporator. The procedure was repeated six times obtaining 3.44 g of dried extract (51.3% w/w). This latter was dissolved in 60 mL of H_2_O and partitioned with ethyl acetate (EtOAc) for three times. The two fractions obtained through this partitioning were dried in rotary evaporator yielding H_2_O fraction (FrW = 3 g) and EtOAc fraction (FrEt = 440 mg). Subsequently, FrEt and FrW were further fractionated by Medium Pressure Liquid Chromatography (MPLC) (Reveleris®, Bȕchi, Switzerland). In particular, FrEt was dissolved in 1.5 mL of MeOH, injected in C18 column (Select C18 30 μm spherical 4 g, Buchi, Switzerland), and eluted with a gradient of H_2_O (solvent A) and MeOH (solvent B) starting from 5% MeOH up to 100% MeOH in 50 min. The flow rate was 5 mL/min. The three detection wavelengths used were λ 220 nm, 256 nm, and 278 nm. The fractions were collected by volume (5 mL each tube), obtaining 50 tubes. Each fraction was dried in rotary evaporator and analyzed by ^1^H NMR, which guided the selection of the fractions containing the metabolite of interest. Subfractions 10 (9.8 mg) e 22 (5.3 mg) showed respectively the presence of 4-hydroxybenzyl alcohol (gastrodigenin) and bis(4-hydroxybenzyl)ether identified by means of both 2D NMR and MS experiments.

FrW (1.5 g) was dissolved in 2 mL of H_2_O, injected in C18 column (Select C18 50 μm spherical 80 g, Bȕchi, Switzerland), and eluted with a gradient of H_2_O (solvent A) and MeOH (solvent B). The gradient was composed of an isocratic phase of 4.6 min (95% A and 5% B), a gradient to 10% B in 4.6 min, an isocratic phase of 4.6 min (10% B), a gradient 20% B in 4.6 min, an isocratic phase of 4.6 min (20% B), a gradient 30% B in 4.6 min, an isocratic phase of 4.6 min (30% B), a gradient from 100% B in 18.4 min and an isocratic phase of 9.2 min (100% B). The flow rate was 30 mL/min and the run length was 60 min. The three detection wavelengths used were λ 220 nm, 256 nm, and 278 nm. The fractions were collected by volume (25 mL each tube) obtaining 72 tubes. Analogously to FrEt, NMR was employed to guide the selection of the subfraction of interest, namely subfraction 9 (10.3 mg), yielding parishin E, and subfraction 38 (3 mg), yielding parishin A, both elucidated by means of NMR and MS experiments.

### Mass spectrometry

UHPLC-UV–MS analysis was run on a Waters ACQUITY ARC UHPLC/MS system consisting of a QDa mass spectrometer equipped with an electrospray ionization interface and a 2489 UV/Vis detector. The detected wavelengths (λ) were 268 nm and 370 nm. The analyses were performed on a XBridge BEH C18 column (100 × 2.1 mm i.d.; particle size 2.5 μm) with a XBridge BEH C18 VanGuard Cartridge precolumn (5 mm × 2.1 mm i.d.; particle size 1.8 µm). The mobile phases were H_2_O (0.1% formic acid) (A) and MeCN (0.1% formic acid) (B). Gradient: 0–0.78 min, 5% B; 0.78–10.00 min, 5 − 50% B; 10.00–11.00 min, 50–95% B; 11.00–12.00 min, 95% B; 12.00–13.00 min, 95–5% B; 13.00–14.50 min, 5% B. Flow rate: 0.8 mL/min. Injection volume: 4 μL. Electrospray ionization (ESI) in positive and negative mode was applied in the mass scan range of 50–1200 Da. Raw extracts were injected at a concentration of 10 mg/mL, while pre-purified fractions were injected a concentration of 1 mg/mL.

To obtain the exact molecular weight, FrEt subfractions 10 and 22, and FrW subtraction 38 were diluted to 1 µg/mL and analysed in a Xevo G2-XS QTof system through direct infusion. ESI in positive and negative modes was applied in the mass scan range of 50–1200 m/z. ESI source conditions were as follows: capillary = 3 kV, cone = 30 V, source temperature = 120 °C, desolvation temperature = 600 °C, cone gas flow = 50 L/h, and desolvation gas flow = 1 L/h.

### Structure elucidation

#### 4-hydroxybenzyl alcohol (gastrodigenin)

^1^H NMR (600 MHz, D_2_O): *δ* 7.22 (d, 2H, *J* = 8.46 Hz, H2, H6), 6.83 (d, 2H, *J* = 8.46 Hz, H3, H5), 4.46 (s, 2H, H7a, H7b).

^13^C NMR: *δ* 155.23 (C4), 132.29 (C1), 129.52 (C2, C6), 115.23 (C3, C5), 63.48 (C7).

QToF-MS: *m/z* 107.0486 [M-H_2_O + H]^+^

#### bis(4-hydroxybenzyl)ether

^1^H NMR (600 MHz, CD_3_OD): *δ* 7.15 (d, 2H, *J* = 8.50 Hz, H2, H6), 6.76 (d, 2H, *J* = 8.50 Hz, H3, H5), 4.39 (s, 2H, H7a, H7b).

^13^C NMR: *δ* 156.87 (C4), 128.74 (C1), 129.66 (C2, C6), 114,75 (C3, C5), 71.20 (C7).

QToF-MS: *m/z* 229.0870 [M-H]^−^, *m/z* 123.0450 [M/2-H]^−^.

#### Parishin A

^1^H-NMR (600 MHz, CD_3_OD:D_2_O 50:50): *δ* 7.23 (4H, d, *J* = 8.98 Hz, H-2, 2", 6, 6"); 7.06 (2H, d, *J* = 8.98 Hz, H-2', 6'); 7.04 (4H, d, *J* = 8.98 Hz, H-3, 3", 5, 5"); 6.99 (2H, d, *J* = 8.98 Hz, H-3', 5'); 4.92 (4H, d, H-7, 7"); 4.89 (2H, d, H-8, 8"); 4.77 (1H, d, H-8'); 4.76 (2H, d, H-7'); 3.80 (6H, dd, *J*_*1*_ = 1.96 Hz, *J*_*2*_ = 12.50 Hz, H-13a, 13'a, 13"a); 3.68 (6H, dd, *J*_*1*_ = 5.60 Hz, *J*_*2*_ = 12.50 Hz, H-13b, 13'b, 13"b); 3.48 (3H, overlapping, H-10, 10', 10"); 3.46 (3H, overlapping, H-9, 9', 9"); 3.41 (3H, overlapping, H-11, 11', 11"); 3.32 (3H, overlapping, H-12, 12', 12"); 2.93 (2H, d, *J* = 15.3 Hz, H-15a, 15a"); 2.74 (2H, d, *J* = 15.4 Hz, H-15b, 15b").

^13^C-NMR: *δ* 173.3 (C-14’); 170.3 (C-14, 14"); 157.3 (C-4, 4', 4"); *δ* 130.1 (C-2, 2’, 2’’, 6, 6’, 6’’); 130.03 (C-1, 1’, 1’’); 116.4 (C-3, 3’’, 5, 5"); 116.3 (C-3', 5'); 102.6 (C-8'); 100.3 (C-8, 8"); 76.30(C-12, 12', 12"); 75.9 (C-10, 10', 10"); 73.5 (C-15'); 73.12 (C-9, 9', 9"); 69.2 (C-11, 11', 11"); 66.9 (C-7'); 66.4 (C-7,7"); 60.6 (C-13, 13', 13"); 43.3 (C-15, 15").

QToF-MS: *m/z* 1041.3093 [M + HCOO]^−^, *m/z* 995.30483 [M-H]^−^.

#### Parishin E

^1^H-NMR (600 MHz, D_2_O): *δ* 7.42 (d, 2H, *J* = 8.70 Hz, H2, H6), 7.14 (d, 2H, *J* = 8.70 Hz, H3, H5), 5.15 (d, 1H, *J* = 7.57 Hz, H8), 5.12 (d, 2H, *J* = 2.20 Hz, H7a, H7b), 4.20 (H9), 3.91 (H13a), 3.74 (H13b), 3.7 (H10), 3.6 (H11), 3.6 (H12), 2.94 (d, 1H, *J* = 15.20 Hz, H15a), 2.80 (d, 1H, *J* = 15.20 Hz, H15b), 2.69 (d, 1H, *J* = 15.20 Hz, H17a), 2.58 (d, 1H, *J* = 15.20 Hz, H17b).

^13^C NMR: *δ* 179.47 (C19), 177.49 (C18), 171.1 (C14), 156.33 (C4), 134.6 (C1), 130.04 (C2, C6), 116.62 (C3, C5), 100.31 (C8), 81.5 (C12), 76.8 (C10), 74.24 (C16), 73.4 (C9), 71.5 (C11), 66.27 (C7), 60.49 (C13), 44.9 (C17), 43.1 (C15).

UHPLC-MS: *m/z* 478 [M + NH_4_]^+^, *m/z* 459 [M-H]^−^

## Supplementary Information

Additional file 1.

## Data Availability

The dataset and the NMR spectra generated during the current study are available in the Zenodo repository (10.5281/zenodo.14960480).
