# Supplementary material for: Chemoprofiling of Himantoglossum robertianum (Loisel.) P. Delforge leaves reveals predominance of gastrodigenin and structurally related compounds
Source: Nat Prod Bioprospect. 2025 Jul 16;15(1):45. doi: 10.1007/s13659-025-00526-7 (PMC12267805; doi:10.1007/s13659-025-00526-7)
Supplement: Supplementary file 1 — Additional file 1. [file 13659_2025_526_MOESM1_ESM.docx]

**Chemoprofiling of *Himantoglossum robertianum* (Loisel.) P. Delforge leaves reveals predominance of gastrodigenin and structurally related compounds**

Ilaria Chiocchio,^§a^ Antonio De Agostini,^§b^ Manuela Mandrone,*^a^ Pierluigi Cortis,^b^ Clarissa Tarozzi,^a^ Ferruccio Poli,^a^ Cinzia Sanna^b^

^§^Co-first authors

^a^Department of Pharmacy and Biotechnology (FaBit), Alma Mater Studiorum, University of Bologna, Via Irnerio 42, 40126, Bologna, Italy.

^b^Department of Life and Environmental Sciences, University of Cagliari, Via S. Ignazio da Laconi 13, 09123 Cagliari, Italy.

^*^ Correspondence: Manuela Mandrone

+390512091294 (office)

email: [manuela.mandrone2@unibo.it](mailto:manuela.mandrone2@unibo.it)


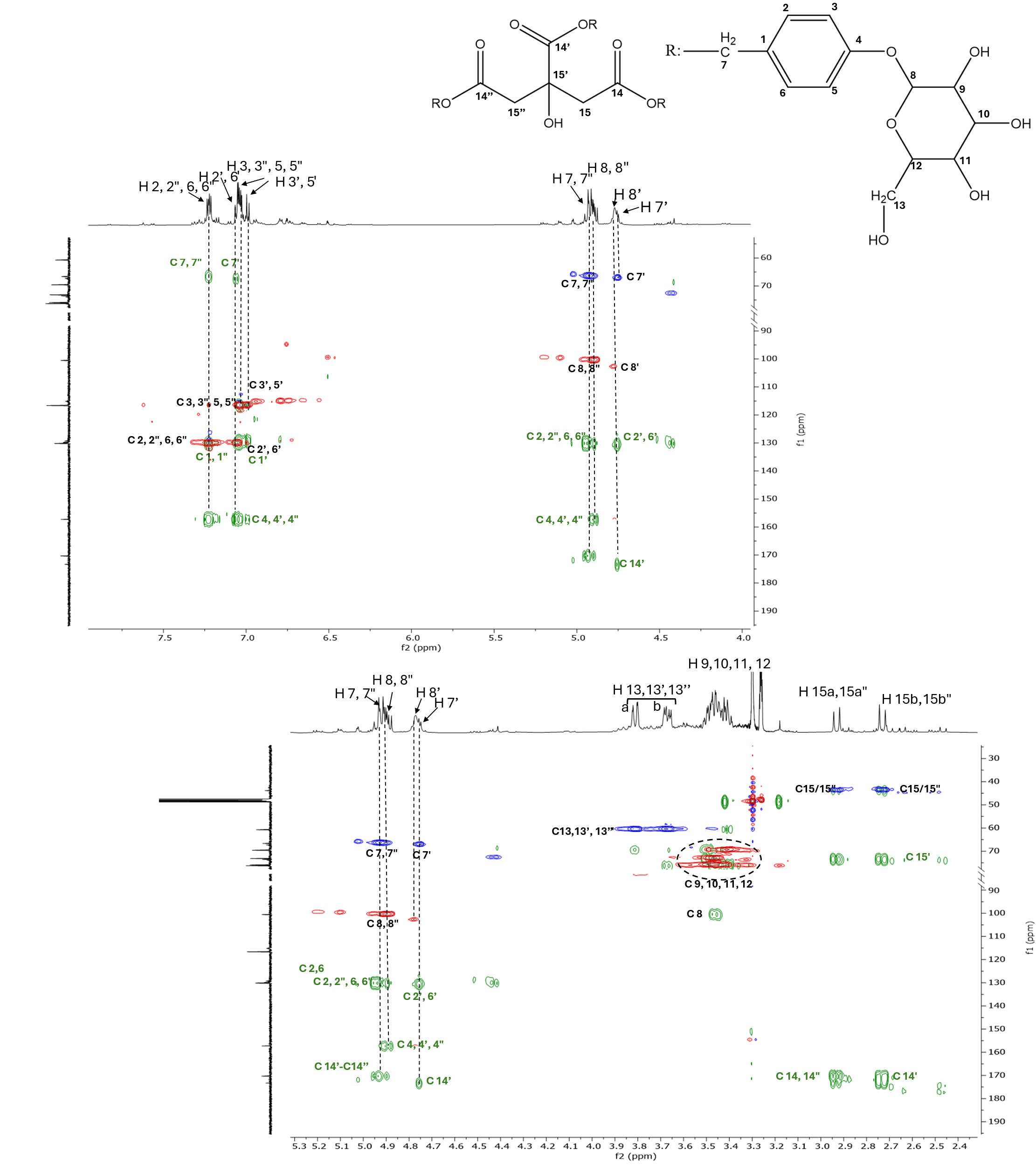


**Figure S1**. Heteronuclear correlations in parishin A by means of HMBC and HSQC NMR. Green dots represent HMBC correlations, blue and red dots represent HSQC correlations.


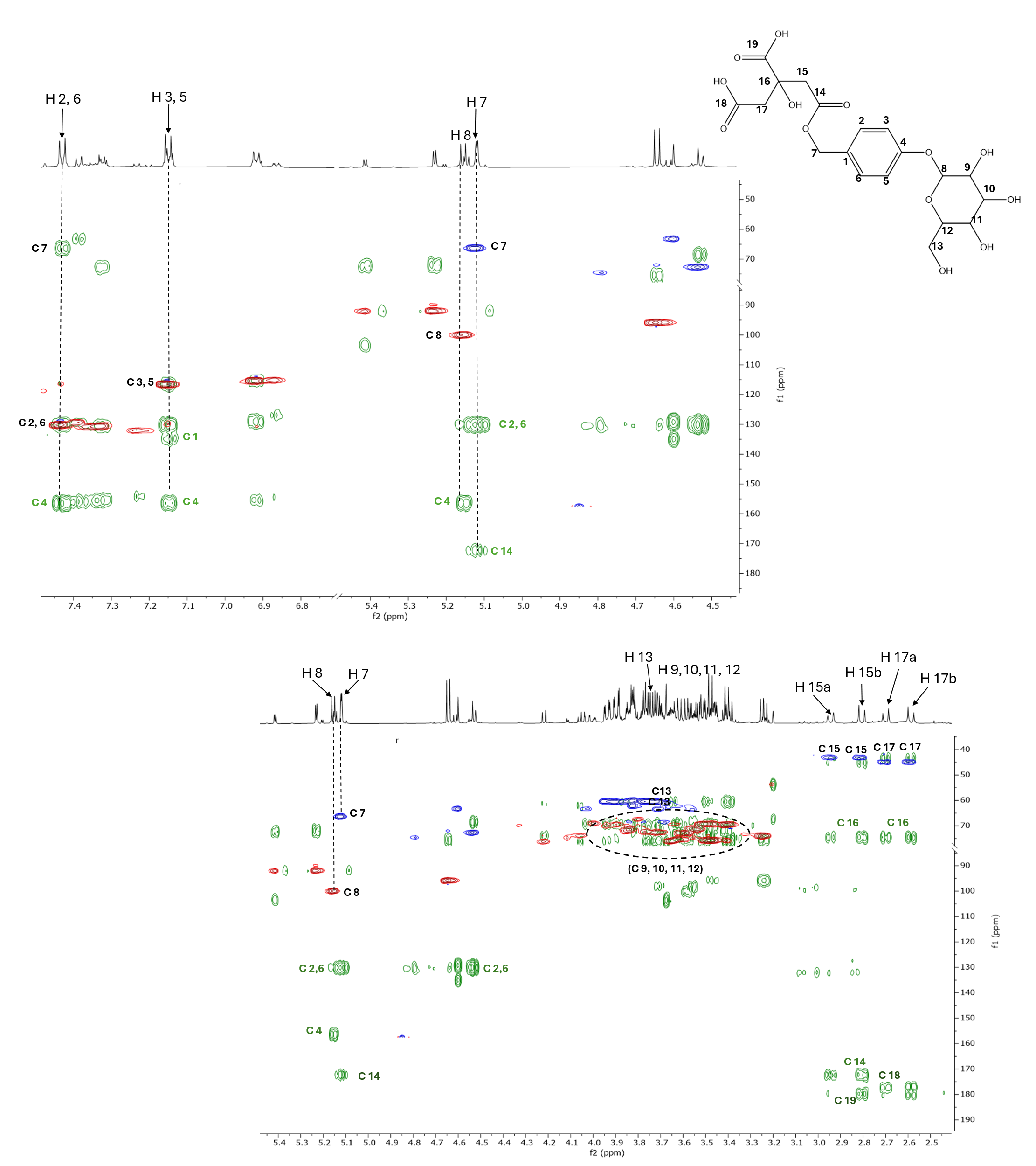


**Figure S2**. Heteronuclear correlations in parishin E by means of HMBC and HSQC NMR. Green dots represent HMBC correlations, blue and red dots represent HSQC correlations.


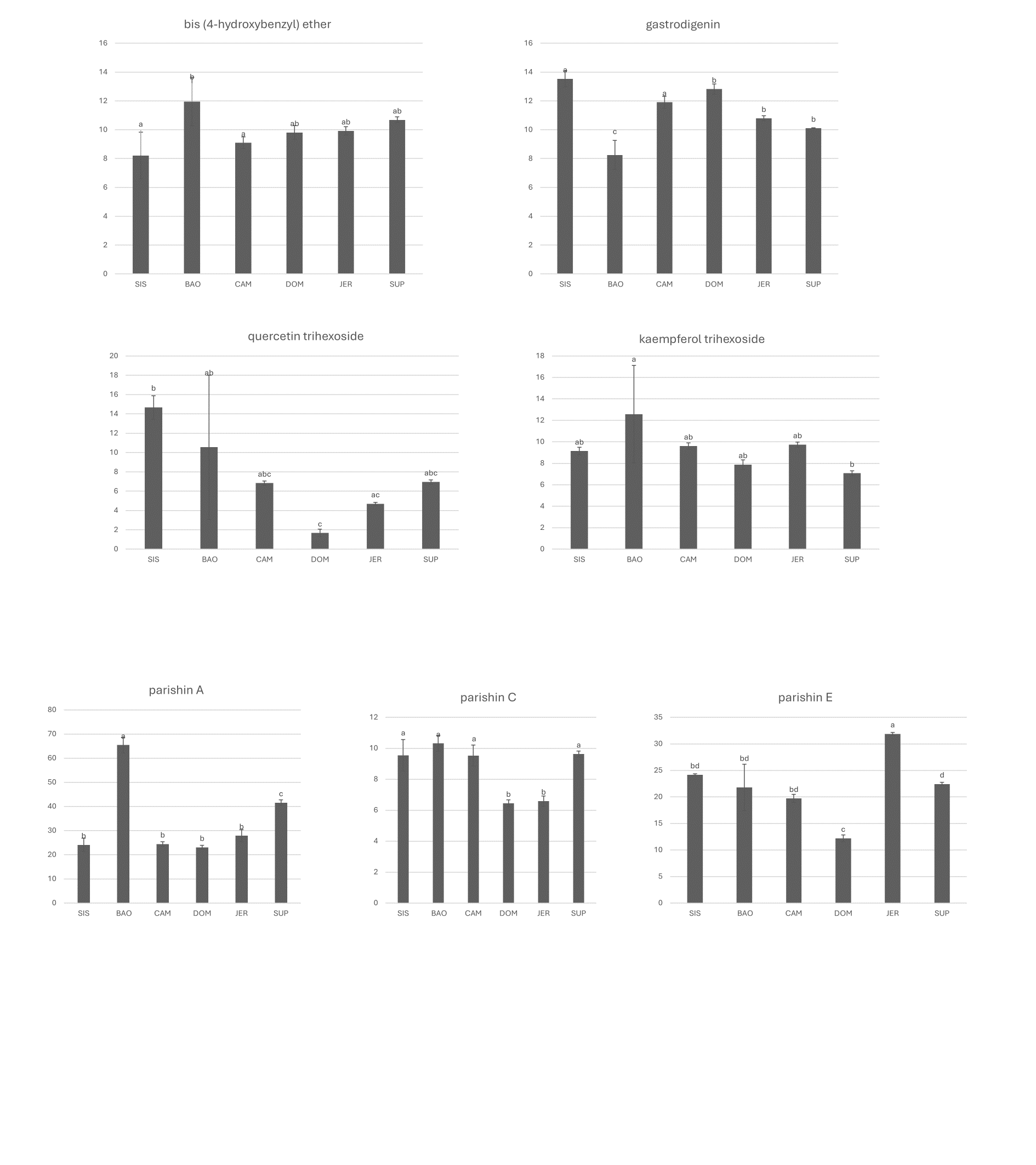


**Figure S3**. Histograms representing the concentration of specialized metabolites for each sampling location expressed in μg of metabolite for mg of leaves (DW). Different letters indicate significant differences in ANOVA test (*p* <0.05).
